# Serum C-reactive protein metabolite (CRPM) is associated with incidence of contralateral knee osteoarthritis

**DOI:** 10.1038/s41598-021-86064-x

**Published:** 2021-03-22

**Authors:** Anne-Christine Bay-Jensen, Asger Bihlet, Inger Byrjalsen, Jeppe Ragnar Andersen, Bente Juhl Riis, Claus Christiansen, Martin Michaelis, Hans Guehring, Christoph Ladel, Morten A. Karsdal

**Affiliations:** 1 Immuno-Science, Nordic Bioscience, Biomarkers and Research, Herlev Hovedgade, 2730 Herlev, Denmark; 2 NBCD A/S, Herlev Hovedgade 82, 2730 Herlev, Denmark; 3 Nordic Bioscience, Clinical Development, Herlev Hovedgade 82, 2730 Herlev, Denmark; 4 Merck KGaA, Translational Innovation Platform Immunology, Frankfurter Strasse 250, 64293 Darmstadt, Germany; 5 Merck KGaA, Global Patient Safety, Frankfurter Strasse 250, 64293 Darmstadt, Germany; 6 BioBone B.V., Amsterdam, Netherlands

**Keywords:** Analytical biochemistry, Immunological techniques, Biological techniques, Biotechnology, Immunology, Biomarkers, Rheumatology, Enzyme mechanisms, Proteases, Proteins

## Abstract

The heterogeneous nature of osteoarthritis (OA) and the need to subtype patients is widely accepted in the field. The biomarker CRPM, a metabolite of C-reactive protein (CRP), is released to the circulation during inflammation. Blood CRPM levels have shown to be associated with disease activity and response to treatment in rheumatoid arthritis (RA). We investigated the level of blood CRPM in OA compared to RA using data from two phase III knee OA and two RA studies (N = 1591). Moreover, the association between CRPM levels and radiographic progression was investigated. The mean CRPM levels were significantly lower in OA (8.5 [95% CI 8.3–8.8] ng/mL, n = 781) compared to the RA patients (12.8 [9.5–16.0] ng/mL, n = 60); however, a significant subset of OA patients (31%) had CRPM levels (≥ 9 ng/mL) comparable to RA. Furthermore, OA patients (n = 152) with CRPM levels ≥ 9 ng/mL were more likely to develop contra-lateral knee OA assessed by X-ray over a two-year follow-up period with an odds ratio of 2.2 [1.0–4.7]. These data suggest that CRPM is a blood-based biochemical marker for early identification OA patients with an inflammatory phenotype.

## Introduction

Osteoarthritis (OA) is a heterogeneous, painful and serious disease. The heterogeneity may be attributed to the existence of several phenotypes and underlining molecular subtypes also called endotypes. These phenotypes are not fully characterized, however it has been suggested that presence of systemic and/or local inflammation may be descriptive of one phenotypes^1,2^. The lack of understanding of OA phenotypes may be a major limitation for interventional trials, as patients with different phenotypes are expected to respond differently to a given drug. This limitation may be one of the main reasons for the lack of approved disease modifying drugs in OA (DMOADs). Anti-inflammatory treatment options (anti-TNF-alpha and anti-IL-1R1)^3–5^ have been tested interventional trial including patient populations with defined OA, however with no or limited description to their phenotype that may indicate differential response across different sub-populations. It has been suggested that a drug like lutikizumab (monoclonal antibody against IL1α/β) would work in patients with an inflammatory phenotype, while sprifermin (recombinant and truncated form of fibroblast growth factor 18) would work in patients with deficit of cartilage repair endotype^6–8^. There is a clear medical need to identify phenotypes in OA to enable development of more efficacious drugs.

Biochemical, blood-based and objective markers could be attractive tools for identifying sub-populations in OA; however, to date there are not such marker available for diagnostic use. Acute inflammation can be quantified in blood by the acute phase reactant C-reactive protein (CRP). CRP is highly elevated in rheumatic disorders such as rheumatoid arthritis (RA)^9^, where it is being used as part of the ACR-EULAR diagnosis criteria^10^. Blood CRP level have been tested in several studies of OA^11–13^, however with differential results and with no clear conclusion to its applicability as a diagnostic marker in OA. One explanation may be the large biological variation associated with the role of CRP as an acute reactant. CRP is mainly expressed and released from the liver in response to injury and infection, where from it is released in its pentameric form and binds to the site of injury followed by binding to complement and FcR^14^. CRP is metabolized by proteases, such as matrix metalloproteinases (MMPs), resulting in the release of the CRP metabolites. One such metabolite is the MMP degradation product CRPM^15^. Serum CRPM levels have been shown to be higher in subjects with radiographic knee OA^16^, and to be dose- and time-dependently inhibited in response to anti-TNF and anti-IL-6 treatment in RA^17,18^. In addition, in RA CRPM have been shown to be significantly correlated with disease activity measures such as DAS28, CDAI, SDAI, HAQ, ESR and CRP^18,19^.

The primary hypothesis of the study, was that a notable proportion of OA patients have inflammation levels comparable to that of RA patients, as quantified by CRP or CRPM. The second hypothesis is that one of such inflammatory reactants would be associated with progression, and thus possibly reflect and inflammatory endotype with a distinctive clinical prognosis.

## Results

### Cohort and sub-group description

There were more females in the moderate to severe (MS) RA cohort (*p* = 0.031) and significantly more Caucasians in the OA cohort (*p* = 0.023) as compared to E-RA cohort (Table 1). Mean age was significantly higher in the OA cohort compared to E-RA (*p* < 0.0001) (Table 1). There was no difference in BMI between the MS-RA and OA cohort. BMI was not recorded in the E-RA cohort. DAS28 was higher in the MS-RA cohort compared to the E-RA cohort (*p* < 0.0001). Both the MS-RA and the OA cohorts had symptomatic and radiographic disease as observed by DAS28, WOMAC total and radiographic severity scores. There was no difference between the OA-ALL cohort and the OA-CC cohort (Table 1).

**Table 1:** Cohort descriptions including subjects with serum CRP and CRPM measurement available at baseline.

	E-RA (reference)	MS-RA	Difference to E-AR. *p*-value	OA-ALL	Difference to E-AR. *p*-value	OA-CC	Difference to OA-ALL
N (% females)	60 (70)	598 (83)	0.031	781 (64)	ns	152 (56)	ns
Number of Caucasian (%)	44 (73)	429 (72)	ns	675 (86)	0.023	140 (92)	ns
Age, years	53.0 (40.5–61.0)	53.0 (44.0–61.0)	ns	64.1 (60.4–69.0)	< 0.0001	62.9 (58.7–67.6)	ns
BMI, Kg/m2	–	26.5 (23.1–30.4)	–	28.0 (25.4–31.2)	–	27.4 (24.9–30.5)	ns
DAS28	3.94 (3.05–5.32)	6.53 (5.93–7.18)	< 0.0001	–	–	–	–
WOMAC total	–	–	–	1086 (847–1384)^b^	–	1050 (817–1350)^b^	ns
VAS pain	53.0 (31.5–75.0)	53.0 (41.0–71.0)	ns	50.0 (35.0–65.0)^b^	ns	49.5 (35.0–64.5)^b^	ns
Radiographic severity	–	12.3 (4.4–24.0)^a^	–	2.0 (2.0–2.0)^b^	–	2.0 (2.0–2.0)^b^	ns
CRP, mg/L	6.4 (1.7–15.4)	13.3 (5.8–28.2)^c^	< 0.0001	1.7 (0.9–3.3)	< 0.0001	1.7 (0.8–3.4)	ns
CRPM, ng/mL	12.8 (9.5–16.0)	15.5 (12.0–20.1)	ns	8.0 (6.3–9.9)	< 0.0001	8.2 (6.2–9.7)	ns

Data is shown as numbers (%) or median (IQR). Differences between cohorts were tested with Mann–Whitney test or Chi-squared test.

*E-RA* early RA cohort, *MS-RA* moderate-severe RA, *OA-ALL* the full OA cohort, *OA-CC* the case–control cohort, *DAS28* disease activity score 28, *VAS* visual analog score, *KL* Kellgren-Lawrence grade.

^a^RA erosion score.

^b^WOMAC total, VAS pain and KL grade of the signal knee.

^c^Adjusted from mg/dL to mg/L.

### Levels of serum CRPM and CRP in rheumatoid arthritis and osteoarthritis

**Figure 1:**
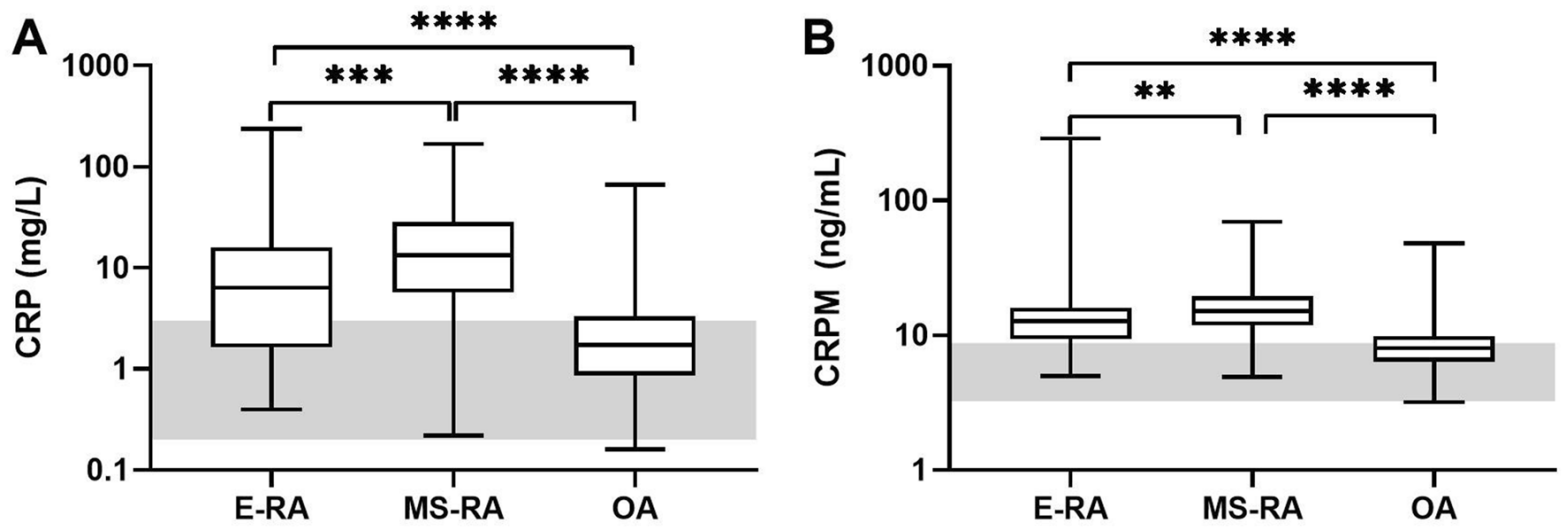
Baseline serum CRP and CRPM levels in early arthritis (E-RA), moderate to severe RA (MS-RA) and osteoarthritis (OA) patients compared to early RA patients. (**A**) Serum CRP levels and (**B**) serum CRPM levels. Grey vertical bars indicate the normal references ranges established in the central lab measuring the markers. Data are shown as IQR and differences are tested by ANCOVA using log transformed biomarker data adjusting for covariates (age, gender, race, VAS pain). Bonferroni corrected significance levels: **p < 0.01 and ****p < 0.0001.

The measured concentrations of serum CRPM and CRP are seen in Table 1 and in Fig. 1. The levels of both CRP and CRPM were marginally higher in the OA cohort as compared to the central lab established reference levels (Fig. 1). Log CRP was statistical significantly correlated to age, BMI, gender and VAS pain (Table 2). Log CRPM was correlated to age, gender and VAS pain, but not BMI (Table 2). Both mean log CRP and log CRPM were significantly lower in Caucasians and in females (Table 2). CRP and CRPM was correlated only in RA and not in OA (Fig. S1).

**Table 2:** The univariate association of log CRP and log CRPM levels with common age, BMI, gender, VAS pain, gender, race or each other, and shown as either univariate Pearson correlation coefficient *r* or difference (SD) in mean levels.

		Log CRP	Log CRPM
Age	*r* *p*	− 0.361 < 0.0001	− 0.415 < 0.0001
BMI*	*r* *p*	0.098 0.0003	− 0.039 0.1531
VAS pain	*r* *p*	0.150 < 0.0001	0.135 < 0.0001
Log CRPM	*r* *p*	0.684 < 0.0001	–
Gender (female vs. males)	Difference (SD) *p*	− 0.23 (0.62) < 0.0001	− 0.08 (0.21) < 0.0001
Race (Caucasian vs. others)	Difference (SD) *p*	− 0.21 (0.62) < 0.0001	− 0.14 (0.21) < 0.0001

All cohorts were included in the analysis, except for BMI which was only available in the MS-RA and the OA cohort (*).

**Figure 2:**
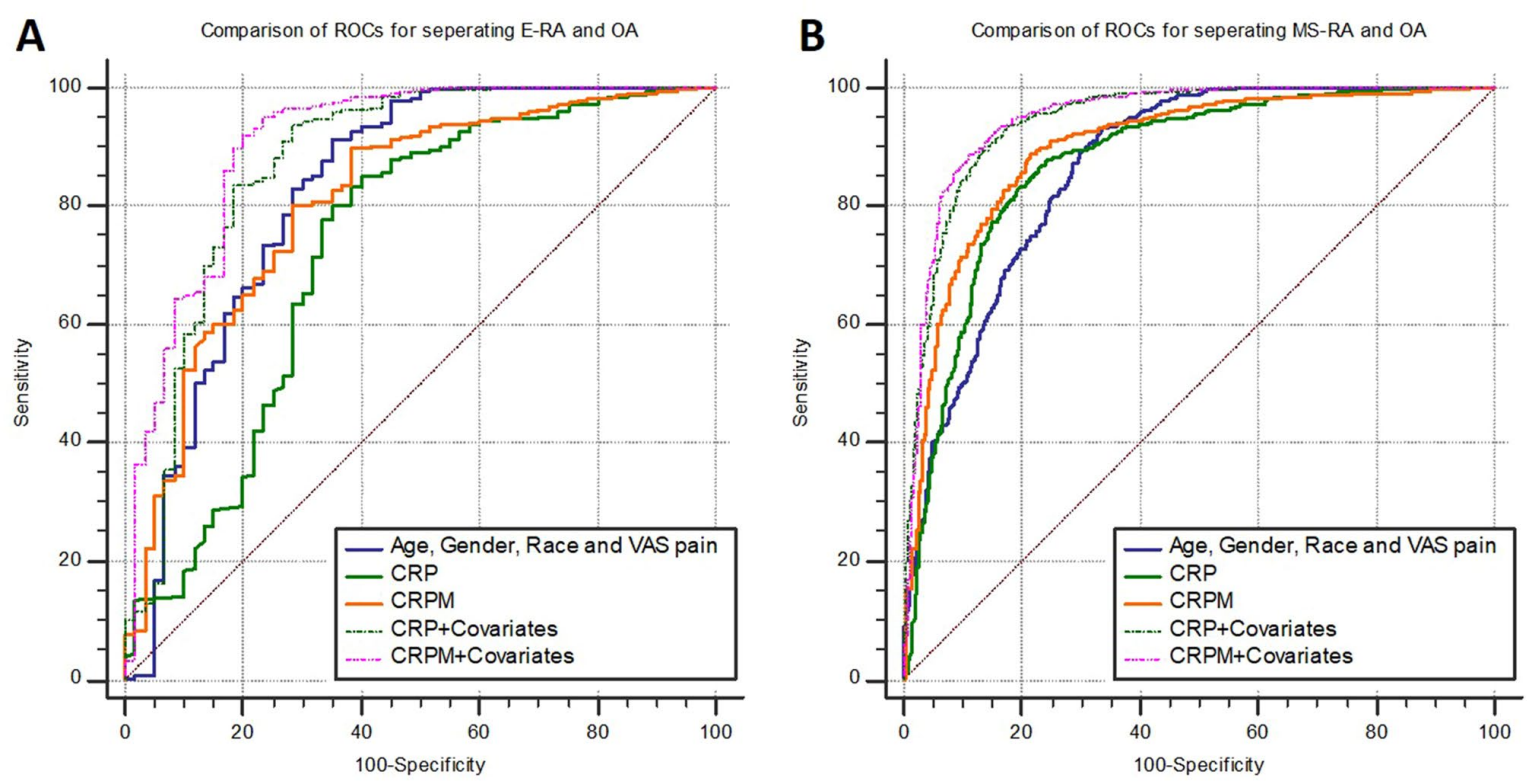
ROC analyses of for separating OA from either E-RA or MS-RA including covariates (Age, gender, race, BMI* and VAS pain) or markers alone or in combination. *BMI was only available for the MS-RA and OA cohorts.

After adjustment for the covariates outlined in Table 2, levels of both serum CRP and serum CRPM were found to be significantly higher in the MS-RA cohort, and significantly lower in the OA cohort as compared to the E-RA cohort (Table 3, supplementary (S) Table S1). The biggest differences were observed between OA and MS-RA for both markers (Table 3). CRP, CRPM and covariates were able to separate OA from E-RA (Fig. 2) with AUCs of 0.73, 0.81 and 0.83, and OA from MS-RA with AUCs of 0.88, 0.90 and 0.87 (Table 3, Fig. 2). None of the markers performed better than the covariates in separating OA from E-RA (Fig. 2A, Table S2), however CRPM was better than the covariates in separating OA from MS-RA with mean difference (SD) in AUC of 0.03 (0.01), *p* = 0.0098 (Fig. 2B, Table S3). CRPM were better at separating OA from E-RA than CRP with an AUC difference (SE) of 0.08 (0.04), *p* = 0.036, and OA from MS-RA 0.02 (0.01), *p* = 0.02 (Fig. 2, Tables S2 and S3). The combined model of either marker and covariates provided a significantly (*p* < 0.01) better separation of OA and E-RA with differences (SE) to covariates alone of 0.05 (0.02) and 0.08 (0.02) CRP and CRPM respectively (Fig. 2A, Table S2), and in separating OA and MS-RA with differences of 0.08 (0.02) for both markers (Fig. 2B, Table S3).

**Table 3:** Estimated marginal mean differences (EMMD) with standard error (SE) and AUCs between the RA cohorts and OA.

		Log CRP	Log CRPM
E-RA	EMMD (SE) *p* value	− 0.47 (0.07) < 0.0001	− 0.19 (0.02) < 0.0001
	AUC_covariates_ [95%-CI] AUC_marker_ [95%-CI] AUC_Marker+ covariates_ [95%-CI]	0.83 [0.80–0.85] 0.73 [0.70–0.76] 0.87 [0.85–0.90]	0.83 [0.80–0.85] 0.81 [0.78–0.84] 0.90 [0.88–0.93]
MS-RA*	EMMD (SE) *p* value	− 0.77 (0.03) < 0.0001	− 0.27 (0.01) < 0.0001
	AUC_covariates_ [95%-CI] AUC_marker_ [95%-CI] AUC_Marker+ covariates_ [95%-CI]	0.87 [0.85–0.89] 0.88 [0.86–0.89] 0.95 [0.93–0.96]	0.87 [0.85–0.89] 0.90 [0.88–0.91] 0.95 [0.94–0.96]

Adjusted for the covariates age, gender, VAS pain and race. *Comparison of the OA with MS-RA cohort was furthermore adjusted for BMI.

### Association of baseline serum CRPM and CRP with OA progression

The second objective was to investigate the level of CRPM in knee OA patients and whether a level was associated with radiographic progression. Different cut-offs to determine whether CRP is high are commonly being applied by labs and rheumatologist around the world used for CRP. We have included these cut-offs alongside with the mathematical derived cut-offs (Table 4). In addition, we use the E-RA cohort as our references and set exploratory cut-offs at the < 25% (low), 25 to 50% (moderate), 50 to 75% (high) and > 75% (very high) quartiles for both CRP and CRPM. More than ninety percent of the MS-RA population had moderate to very high CRP and CRPM levels, confirming the expected that both CRP and CRPM is high in RA patients with moderate to severe disease (Table 4).

**Table 4:** Defining exploratory cut-off for low to very high levels of CRPM and CRP.

	E-RA (n = 60)	MS-RA (n = 598)	OA-ALL (n = 781)
	No. of patients (%)	No. of patients (%)	No. of patients (%)
**CRP**			
Reference level < 3 mg/L	19 (31.7)	76 (12.7)	558 (71.4)
Reference level 3–5 mg/L	10 (10.9)	54 (9.0)	106 (13.6)
Reference level 5–10 mg/L	14 (23.3)	139 (23.2)	75 (9.6)
Reference level ≥ 10 mg/L	22 (36.7)	329 (55.0)	42 (5.4)

	E-RA (n = 60)	MS-RA (n = 598)	OA-ALL (n = 781)
	Interquartile range	No. of patients (%)	No. of patients (%)
**CRP**			
Low	< 2 mg/L	42 (7.0)	382 (48.9)
Moderate	2–6 mg/L	126 (21.1)	315 (40.3)
High	6–14 mg/L	142 (23.7)	54 (6.9)
Very high	≥ 14 mg/L	288 (48.2)	30 (3.8)
**CRPM**			
Low	< 9 ng/mL	58 (9.7)	538 (68.9)
Moderate	9–13 ng/mL	118 (19.7)	181 (23.2)
High	13–16 ng/mL	141 (23.6)	41 (5.2)
Very high	≥ 16 ng/mL	318 (47.0)	21 (2.7)

Twenty-nine percent of the OA patients had CRP levels higher than 3 mg/L, 15.0% had levels higher than 5 mg/L and only 5.4% had levels higher than 10 mg/L (Table 4). Using the quartile range cut-offs then 51.0% of the OA patients had moderate to very high levels of CRP, whereas 31.1% of the OA patients had moderate to very high CRPM levels (Table 4).

**Figure 3:**
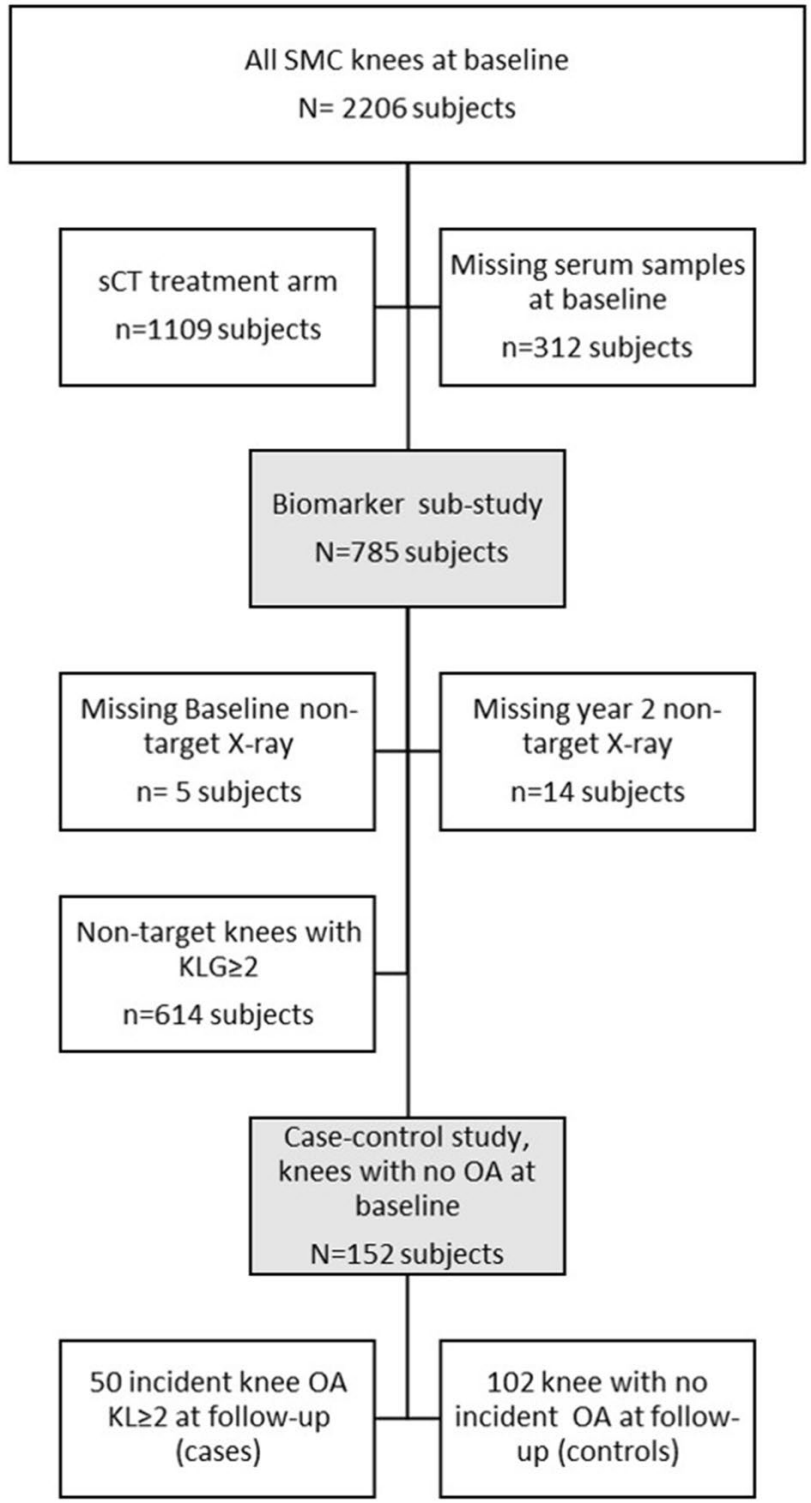
Overview for the osteoarthritis case–control study.

The OA patients from the placebo group with no OA in the contra-lateral knee was divided into cases (n = 50) and controls (n = 102) where cases developed contra-lateral knee OA and controls did not over the two year follow period (see method section and Fig. 3 for more details). This was followed by an analysis of the prognostic ability of each of the markers using the defined cut-offs. Independent of which cut-off was applied for CRP, it was not associated with two-year incidence of contra-lateral knee OA (Table 5). Patients with moderate to very high CRPM (≥ 9 ng/mL, n = 30 control / 20 cases) were 2.2-times more likely to develop OA in the knee with no OA at baseline (Table 5).

**Table 5:** The odd ratio for radiographic progression (2-year KL change ≥ 2) at elevated biomarker levels, after adjustment for baseline BMI, gender, age, JSW, KL and VAS pain, in OA-CC cohort.

	Odds ratio [95% CI] *p* value
CRP ≥ 2 mg/L	1.83 [0.84, 3.99] > 0.1
CRP ≥ 3 mg/L	0.66 [0.28, 1.54] > 0.1
CRP ≥ 5 mg/L	0.75 [0.26, 2.17] > 0.1
CRP ≥ 10 mg/L	0.92 [0.22, 3.94] > 0.1
CRPM ≥ 9 ng/mL	2.18 [1.01, 4.72] 0.048

## Discussion

We hypothesized that an inflammatory biomarker could be associated with an inflammatory endotype associated with progression. Serum levels of CRPM were able to separate OA from RA with AUCs of more than 0.81, underlining that CRPM, as CRP, is highly related with inflammatory condition. However, we also found that more than a third of the OA patients had CRPM and half had CRP levels corresponding to the levels observed in RA, indicating that as significant subset of patients may have an inflammatory signature. Interestingly, only CRPM, and not CRP, was prognostic for progression of OA.

CRPM is a metabolite of C-reactive protein, which is released from the inflamed tissue after being degraded by MMPs released by for example macrophages^20,21^. Both CRP and CRPM are reduced in response to anti-inflammatory treatments such as anti-IL1 or anti-IL-6 receptor antibodies^18,22^. Presently, no subset analyses for identification profiles associated with progression in OA have been presented. In addition, the literature on CRP as a biomarker for both progression and diagnosis of OA is contradicting with both positive and negative observations^23^. This is interesting in respect to disorders like OA, as this is not classically thought of as acute or systemic inflammatory diseases, however a subpart of patients may have an inflammatory endotype that could be associated with progression.

A decade ago, Kerkhof et al.^24^ and Attur et al.^25^, showed that specific genotypes linked with the IL-1β and IL-1 receptor antagonist were associated with radiographic severity of OA. They did not find an association with radiographic progression in this study. These data was further substantiated a year later by Attur et al.^26^, that demonstrated that two distinct subgroups existed amongst OA patients where IL-1β expression was increased as compared to controls. This IL-1β endotype was associated with worse pain and function scores as well as radiographic progression. Recently, Attur et al. further investigated CTA and TTG haplotypes of the *IL1RN* gene and their association with radiographic severity and incident OA^27^. They found that the TTG haplotype was significantly and consistently associated with incident minimal joint space width, radiographic OA (severity) and radiographic progression. Moreover, they found that plasma levels of IL-1Ra were independently associated with OA severity and radiographic progression^28^. In the OAI-FNIH biomarker consortium, Kraus et al. found that blood markers of cartilage and bone remodelling as well as the inflammation-associated marker hyaluronic acid (HA) was associated with radiographic and pain progression^29,30^. Of note, as in our investigation, the OAI study was designed to include both progressors and non-progressors, where all subjects had OA at baseline. These data clearly point in the direction of the existence of an inflammatory endotype in OA, and such endotype could be defined by specific risk associated polymorphism and the level of one or more biochemical or transcriptomics markers that assess inflammatory activity.

Progression in OA in clinical trial settings is commonly defined as changes to joint space width or increase in KLG over a time frame of one to three years. However, drivers of progression most likely have different origins amongst patients^1,31,32^. Thus, we know how the patients feel and function, but we don’t know why and what drive a worsening of disease. This may be key in understanding why certain drugs fail in development: if only 30% of the trial population will benefit from targeting as specific pathway, and with a plausible 50% response rate, the hurdle for documenting a clinical benefit is high, or impossible. Thus, there is a medical need for better subgrouping of patients by developing tools for endotyping. We propose that emphasis should be put on identifying sub-groups or subtypes of OA and to target drug development to each group followed by investigation of the efficacy^33^. We have recently published that CRPM together with other markers of tissue turnover may further subgroup patients into different endotypes, and that these endotypes display different radiographic progression types in RA^34^. Interestingly, we also included OA patients in the analysis that approximately 25% of the OA patients clustered with a subset of RA patients which were primarily in bone and cartilage markers, whereas about 10% of the patients clustered with RA patients that were high in bone, cartilage, macrophage and interstitial matrix turnover markers including CRPM. CRPM is most likely just one signal of many that could be investigated and combined to enable endotyping of patients and thereby better characterization of the patient profiles and planning of appropriate treatment regimen. CRPM has also recently been investigated in idiopathic pulmonary fibrosis^35^ and spondyloarthropathy^36^ and has been associated with progression. This may suggest that there is a general inflammatory endotype associated with progression of chronic tissue diseases, which may be worthwhile investigating for the benefit of patients responding.

A limitation of the OA studies is that all patients have OA at baseline, in contrast to general demographic studies, thus the results cannot be extended to classification of CRPM as diagnostic biomarker (i.e. finding knee OA patients in a risk population). Instead, the analysis is limited to the identification of OA patients likely to develop bilateral and radiographic knee OA. Another limitation is the selection bias which is introduced in a well-defined trial protocol. From a clinical perspective the population was homogeneous, where most subject had KLG 2 and moderate to severe pain; subjects with mild or very severe pain were excluded, and thus the variation in the dataset is narrow and not representative of all types of patients.

In conclusion, this study provides two major findings: (1) a subset of OA patients appears to have tissue inflammation comparable to that of RA; and (2) high CRPM levels are prognostic of incident knee OA. These data suggest that CRPM is a candidate biomarker of disease activity and for patient profiling. The perspective is to target current bottlenecks in OA drug development and contribute to a stratified medicine approach for more efficacious treatment of OA. There is an urgent medical need for patient stratification in OA; firstly to facilitate better patient recruitment in clinical drug trials and secondly to facilitate better clinical decision-making concerning treatment of OA patients^33,37^.

## Methods

### Patients

#### The early rheumatoid arthritis cohort (E-RA)

Ninety-two early arthritis patients were enrolled in the prospective early arthritis ‘Synoviomics’ cohort at the Academic Medical Center (AMC) in Amsterdam between April 2004 and January 2013 in this study^38^. Of the 92 patients, 60 patients fulfilled the ACR/EULAR 2010 criteria for RA classification^10^ and were selected for current investigations. All patients enrolled in the study had less than one-year disease duration, as measured from the first clinical evidence of joint swelling. Patients had active arthritis in at least one joint and were disease-modifying anti-rheumatic drug (DMARD) naïve. All patients provided written informed consent. The study was performed according to the Declaration of Helsinki and approved by the Medical Ethics Committee of the AMC. Only baseline measures were used in the current study, and demographic data were collected, and the following clinical and laboratory parameters were obtained: serum levels of C-reactive protein (CRP); erythrocyte sedimentation rate (ESR); 68 tender and 66 swollen joint counts (TJC68 and SJC66). Other parameters were recorded; however, this data was not used in current study. The prognostic data was published in 2016 by Maijer KI et al.^19^.

#### The moderate to severe RA cohort (MS-RA)

This is a post-hoc analysis of the available data from the LITHE sub-study, which have previously been thoroughly described by Blair et al. and Bay-Jensen et al.^18,34^. In brief, LITHE is a 2-year phase III, multicentre, randomized, three-arm, placebo-controlled, parallel group trial in patients with moderate to severely active RA who had an inadequate response to MTX^39^. Current analysis only apply baseline data from the biomarker sub-study of LITHE consisting of serum samples from a one year, double-blinded treatment study where 704 patients were randomized 1:1:1 to one of 3 treatment groups: 4 mg/kg or 8 mg/kg TCZ, or placebo (PBO) in combination with a stable dosage of MTX (10–25 mg/week). The clinical study and the biomarker measurements were approved by the ethics committee at each participating institution and was conducted according to the Principles of Good Clinical Practice (GCP) and according to the Declaration of Helsinki. Fasted serum for biomarker research was scheduled to be collected from patients who provided additional informed written consent. Baseline clinical and demographic measures were used in the current study including age, gender, race, DAS28 and erosion score.

#### The osteoarthritis cohort (OA-ALL)

This post-hoc study also include pooled data from two, double-blinded, randomised, placebo-controlled and multi-center phase III clinical trials assessing the efficacy and safety of an oral formulation of 0.8 mg salmon calcitonin in patients with painful and radiographic knee OA (NCT00486434 (trial 1) and NCT00704847 (trial 2))^40^. The trials were conducted in accordance with the Helsinki Declaration and ICH GCP, and were approved by all applicable Independent Review Boards, Ethics Committees, and regulatory bodies. Each independent trial recruited patients aged 51–80 years with painful OA in the target knee, defined as a Visual Analogue Score of ≥ 150 mm on the Western Ontario and McMasters Osteoarthritis Index (WOMAC) pain subscale (500 mm being the maximum score). In study 2, patients scoring ≤ 150 mm on the pain sub-score could participate if they also scored ≥ 510 mm on the WOMAC function sub-scale (1700 mm being the maximum score). The radiographic inclusion criteria for target knees included Kellgren-Lawrence grades (KLG) 2 or 3, and a Joint Space Width (JSW) of ≥ 2.0 mm. A total of 2,206 patients, corresponding to 4430 knees, were recruited at 19 sites in 11 countries. Patients were followed for two years with regular clinic visits. Details regarding trial design and results are published elsewhere^40^. Fasted serum samples were collected and assessed for biomarker at baseline, 3, 6, 12 and 24 months, however in current study only baseline biomarker data were included.

#### Definitions of OA radiographic progression case-controls (OA-CC)

The case–control (CC) was designed with inspiration from the biomarker analyses conducted by Kraus et al. on data from the OAI-FNIH biomarker consortium^30^. The CC study only included knees with no marked (KLG < 2) radiographic knee OA at baseline. After excluding the sCT treatment arm and patients with no baseline serum samples, the biomarker sub-population consisted of 785 subjects (Fig. 3). Five subjects at baseline and 14 at year two had no radiographic scores (X-ray) recorded. 614 subjects had KLG equal to or greater than two at baseline and therefore had radiographic OA in the non-target knee. A total of 152 subjects were included in the case–control study. Cases were defined as those subjects who progressed to KLG ≥ 2 in the non-target knee at year 2, while controls were those that did not progress in the non-target knee (KLG < 2) (Fig. 3). The case–control study included knees with not radiographic OA at baseline.

### Biomarker measurements

Serum CRPM were measured by a competitive, solid-phase ELISA^41^ according to the manufacture protocol. Assessments were performed by Nordic Bioscience Laboratory, a College of American Pathologists (CAP) certified division of Nordic Bioscience. Assays were run blinded and quality controlled according to internal standard operating procedure. CRP was measured on by ADVIA chemistry CardioPhase High sensitivity C-reactive protein assay (Siemens Healthineers, NY, US) at the Nordic Bioscience Laboratory. Briefly, CRPM is competitive ELISA utilizing a monoclonal antibody. The assay was validated prior to use on the samples including test of serum parallelism and minimum required dilution (MRD). Samples were run in fourfold dilution (MRD) and in duplicates. Samples with duplicate CVs above 15% were rerun; both intra- and inter-assay variation were below < 15%. Failure of 3 out of 5 internal plate controls (2 kit controls and 3 QC controls) also lead to rerun of whole ELISA plate. Samples outside the quantification range were either diluted and rerun or given the value of the lower quantification limit. Batch control was completed using 10 serum samples and master calibrators covering the linear range of the calibration curve.

### Statistics

Summary statistics of general demographics, baseline subject characteristics and biomarker levels was depicted by median and interquartile range (IQR). The number of patients, number of females and Caucasians was summarised by absolute numbers and percentage. Difference between cohorts were analysed by chi-squared, Mann–Whitney or Kruskal–Wallis test with correction for multiple comparison (Dunn’s correction).

Levels in the biomarkers in the different cohorts were compared using ANCOVA using log transformed biomarker data adjusting for covariates (age, gender, race, VAS pain and BMI). Estimated marginal mean differences (EMMD) is to access group differences. Receiver operating curves (ROCs) were used to investigate the level of separation between the OA and RA cohorts and depicted by area under the curves (AUCs). No cross-validation was conducted for verification of the AUCs, due to the exploratory nature of the study design. Baseline univariate linear correlations were assessed after log transformation of the biomarker data and is depicted as Pearson’s R^2^ and linear slope (b).

Odds ratios were calculated by multivariate logistic regression adjusting for age, gender, BMI, baseline JSW, baseline KL grade and baseline VAS pain using the exploratory cut-off values. Because these analyses were exploratory, no adjustment was applied for multiple testing. *P* < 0.05 was considered statistically significant and *p* values < 0.1 were depicted. No imputation was made for missing values, as the percentage of missing values for lack of serum volume was similar among the three groups at all time points. All statistical analyses were conducted MedCalc version 19.2.1. Graphing was performed using Prism GraphPad version 5.03. The analyses follow classical biostatistical methodologies and no new models or algorithms were developed or tested in described analysis, that would need further approval from regulatory authorities.

### Ethics approval and consent to participate

Current investigation applies pseudo-anonymized and retrospective data, collected as part of the original study designs. No new raw data was generated as course of described work. All analyses conducted was post-hoc analysis of existing raw data from the individual clinical studies. Experimental design and use of data was approved by the IRB at Nordic Bioscience. The original clinical study designs, where from the raw data for the post hoc analysis was acquired, were approved by local authorities and with patient informed consent. Specifically, for E-RA: All patients provided written informed consent for blood samples to be drawn and biomarker measurement (data of these) to be used for research identifying diagnostics. All patients provided written informed consent for blood samples to be drawn and biomarker measurement (data) to be used for research use only. Samples were analysed and data management were conducted under GCP (https://www.ema.europa.eu/en/human-regulatory/research-development/compliance/good-clinical-practice). All studies and post-hoc analyses were performed according to the Declaration of Helsinki. Data are stored under current regulation on GDPR, and auditable by the internal DPO and the Danish agency for data (Datatilsynet).

## Supplementary information

Supplementary information.

## Data Availability

The datasets generated and/or analyzed during the current study are not publicly available due to GDPR policies at the respective institutions but are available from the corresponding author on reasonable request. That is; anonymized biomarker data of this study may be available to access. Proposals should be directed to the corresponding author. Access will be provided to researches after the proposal has been reviewed by the IRB and after ethical approvals have been acquired. Samples from the patients are no longer available.

## Abbreviations

AUC: Area under the curve
CC: Case–control
CRP: C-reactive protein
DAS28: Disease activity score 28
DMOAD: Disease modifying OA drug
E: Early
HAQ: Health assessment questionnaire
IL: Interleukin
IQR: Interquartile range
JSW: Joint space width
OA: Osteoarthritis
MMP: Matrix metalloproteinase
MS: Moderate to severe
RA: Rheumatoid arthritis
TNF: Tumour necrosis factor
VAS: Visual analogue score
