# Supplementary material for: Serum C-reactive protein metabolite (CRPM) is associated with incidence of contralateral knee osteoarthritis
Source: Sci Rep. 2021 Mar 22;11:6583. doi: 10.1038/s41598-021-86064-x (PMC7985384; doi:10.1038/s41598-021-86064-x)
Supplement: Supplementary file 1 — Supplementary information. [file 41598_2021_86064_MOESM1_ESM.pdf]

Serum C-reactive protein metabolite (CRPM) is associated incidence of contralateral knee osteoarthritis.

Authors:

1. Anne-Christine Bay-Jensen, PhD, Immuno-science, Nordic Bioscience, Biomarkers and research, Herlev Hovedgade 2730 Herlev, Denmark. Phone: +45 4454 7730. E-mail: acbj@nordicbio.com (corresponding author)
2. Asger Bihlet, MSc, NBCD A/S, Herlev Hovedgade 82, 2730 Herlev, Denmark
3. Inger Byrjalsen, DSc, Nordic Bioscience, Clinical Development, Herlev Hovedgade 82, 2730 Herlev, Denmark
4. Jeppe Ragnar Andersen, MSc, NBCD A/S, Herlev Hovedgade 82, 2730 Herlev, Denmark
5. Bente Juhl Riis, MD, NBCD A/S, Herlev Hovedgade 82, 2730 Herlev, Denmark
6. Claus Christiansen, MD, Nordic Bioscience, Clinical Development, Herlev Hovedgade 82, 2730 Herlev, Denmark
7. Martin Michaelis, MD PhD, Merck KGaA, Translational Innovation Platform Immunology, Frankfurter Strasse 250, 64293 Darmstadt
8. Hans Guehring, MD, Merck KGaA, Global Patient Safety, Frankfurter Strasse 250, 64293 Darmstadt
9. Christoph Ladel, PhD, BioBone B.V., Amsterdam, Netherlands
10. Morten A. Karsdal, PhD, Immuno-science, Nordic Bioscience, Biomarkers and research, Herlev Hovedgade 2730 Herlev, Denmark.

SUPPLEMENTARY DATA.

Table S1. Difference in serum CRP and CRPM between cohorts including comparison of E-RA and MS-RA.

Supplementary to table 3 of the main manuscript. Differences are shown as estimated marginal mean differences (EMMD) with standard errors (SE) and area under the curve (AUC) with 95%-CI.

		**log CRP**		**log CRPM**	
		MS-RA	OA	MS-RA	OA
**E-RA**	EMMD (SE)	0.28 (0.07)	-0.47 (0.07)	0.07 (0.02)	-0.19 (0.02)
	p value	0.0001	< 0.0001	0.0038	< 0.0001
	AUC_{marker} [95%-CI]	0.65 [0.61-0.69]	0.73 [0.70-0.76]	0.64 [0.60-0.67]	0.81 [0.78-0.84]
	$AUC_{covariates}$ [95%-CI]	0.65 [0.61-0.69]	0.83 [0.80-0.85]	0.65 [0.61-0.69]	0.83 [0.80-0.85]
	$AUC_{marker+covariates}$ [95%-CI]	0.70 [0.67-0.74]	0.87 [0.85-0.90]	0.70 [0.66-0.73]	0.90 [0.88-0.93]
**MS-RA**	EMMD (SE)	-	-0.77 (0.03)	-	-0.27 (0.01)
	p value		< 0.0001		< 0.0001
	AUC_{marker} [95%-CI]		0.88 [0.86-0.89]		0.90 [0.88-0.91]
	$AUC_{covariates}$ [95%-CI]		0.87 [0.85-0.89]		0.87 [0.85-0.89]
	$AUC_{marker+covariates}$ [95%-CI]		0.95 [0.93-0.96]		0.95 [0.94-0.96]

Table S2. Difference in area under the curve (AUC) for separating OA from E-RA.

Supplementary to figure 3 of the main manuscript. Data is shown as AUC (SE) and p-value. P > 0.05 is shown as ns.

**E-RA vs. OA**	**Covariates**	**CRP**	**CRPM**	**CRP + Covariates**
**CRP**	0.10 (0.06), ns	-	-	-
**CRPM**	0.02 (0.05), ns	0.08 (0.04), 0.036	-	-
**CRP + Covariates**	0.05 (0.02), 0.0079	0.15 (0.05), 0.0021	-	-
**CRPM + Covariates**	0.08 (0.02), 0.0001	-	0.10 (0.03), 0.0050	0.03 (0.02), 0.037

Table S3. Difference in area under the curve (AUC) for separating OA from MS-RA.

Supplementary to figure 3 of the main manuscript. Data is shown as AUC (SE) and p-value. P > 0.05 is shown as ns.

MS-RA vs. OA	Covariates	CRP	CRPM	CRP + Covariates
**CRP**	0. 01 (0.01), ns	-	-	-
**CRPM**	0.03 (0.01), 0.0098	0.02 (0.01), 0.020	-	-
**CRP + Covariates**	0.08 (0.01), <0.0001	0.07 (0.01), <0.0001	-	-
**CRPM + Covariates**	0.08 (0.02), <0.0001	-	0.05 (0.01), <0.0001	0.00 (0.01), ns

Figure S1. Correlations between CRP and CRPM in the RA and OA cohort.

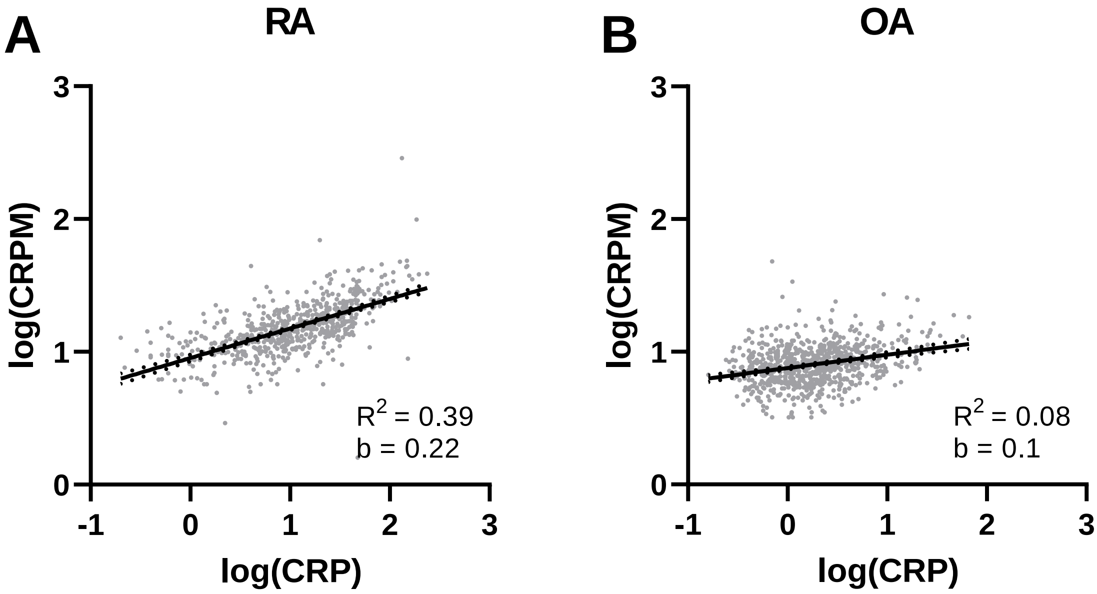

Figure S1. Correlations between serum CRP and CRPM levels in the RA cohorts (A) and the OA cohort (B). Correlations were tested by simple linear regression. Data is shown as individual patients (grey dots and regression line (black line) with 95% confidence interval (black dotted line). Regression coefficient R^2 and slope (b) are given.
